# *Lactobacillus plantarum* displaying CCL3 chemokine in fusion with HIV-1 Gag derived antigen causes increased recruitment of T cells

**DOI:** 10.1186/s12934-015-0360-z

**Published:** 2015-10-22

**Authors:** Katarzyna Kuczkowska, Geir Mathiesen, Vincent G. H. Eijsink, Inger Øynebråten

**Affiliations:** Department of Chemistry, Biotechnology and Food Science, Norwegian University of Life Sciences (NMBU), P.O. Box 5003, 1432 Ås, Norway; Department of Pathology and Centre for Immune Regulation, Oslo University Hospital-Rikshospitalet, and University of Oslo, Oslo, Norway

**Keywords:** Chemokine, CCL3, MIP-1α, Chemotaxis, HIV, Lactic acid bacteria, *Lactobacillus*, Mucosal vaccine

## Abstract

**Background:**

Chemokines are attractive candidates for vaccine adjuvants due to their ability to recruit the immune cells. Lactic acid bacteria (LAB)-based delivery vehicles have potential to be used as a cheap and safe option for vaccination. Chemokine produced on the surface of LAB may potentially enhance the immune response to an antigen and this approach can be considered in development of future mucosal vaccines.

**Results:**

We have constructed strains of *Lactobacillusplantarum* displaying a chemokine on their surface. *L. plantarum* was genetically engineered to express and anchor to the surface a protein called CCL3Gag. CCL3Gag is a fusion protein comprising of truncated HIV-1 Gag antigen and the murine chemokine CCL3, also known as MIP-1α. Various surface anchoring strategies were explored: (1) a lipobox-based covalent membrane anchor, (2) sortase-mediated covalent cell wall anchoring, (3) LysM-based non-covalent cell wall anchoring, and (4) an N-terminal signal peptide-based transmembrane anchor. Protein production and correct localization were confirmed using Western blotting, flow cytometry and immunofluorescence microscopy. Using a chemotaxis assay, we demonstrated that CCL3Gag-producing *L. plantarum* strains are able to recruit immune cells in vitro.

**Conclusions:**

The results show the ability of engineered *L. plantarum* to produce a functional chemotactic protein immobilized on the bacterial surface. We observed that the activity of surface-displayed CCL3Gag differed depending on the type of anchor used. The chemokine which is a part of the bacteria-based vaccine may increase the recruitment of immune cells and, thereby, enhance the reaction of the immune system to the vaccine.

**Electronic supplementary material:**

The online version of this article (doi:10.1186/s12934-015-0360-z) contains supplementary material, which is available to authorized users.

## Background

Chemokines are a group of small (7–12 kDa) proteins important in both homeostatic and inflammatory conditions. They are essential molecules in inflammation and immunity because of their ability to recruit immune cells [1–4]. Based on the arrangement of conserved cysteine residues, chemokines are divided into four different groups: CXC, CC, CX3C, and XC [5]. The tertiary structure of chemokines is highly conserved where the N-terminal domains of chemokines are essential for chemokine receptor activation [6, 7]. Chemokines may organize into dimers or oligomers and there is a large variety in oligomeric forms and in the tendency to oligomerize [7–10]. Because of their ability to recruit the immune cells, chemokines are attractive candidates for vaccine adjuvants and have indeed been used as such with considerable success [11–13].

The chemokine CCL3, also known as macrophage inflammatory protein-1 alpha (MIP-1α) is produced by various types of cells and interacts with CCR1 and CCR5 receptors expressed by dendritic cells (DCs) and macrophages (i.e. antigen-presenting cells), natural killer cells, as well as CD4^+^ and CD8^+^ T cells [14]. It has previously been demonstrated that co-administration of antigens and CCL3 or fusion of antigen to CCL3 increased the antigen-specific CD8^+^ T cell responses to for example influenza and HIV-1 [15–19]. Furthermore, a recent report showed that CCL3 was necessary (but not sufficient) for tumor rejection and efficient DCs migration to lymph nodes [20].

The use of bacteria as vaccine delivery vehicles is a very promising strategy for mucosal vaccination. Non-pathogenic food grade bacteria, in particular lactic acid bacteria (LAB), are promising candidates due to their safe status and the simplicity of genetic engineering [21]. Many species from the *Lactobacillus* genus interact with epithelial cells by binding to pattern recognition receptors (PRRs) [22]. Moreover, it has been shown that some lactobacilli interact with DCs and thereby regulate T cell responses [23]. It is well known that *Lactobacillus* spp. have immunostimulatory properties that may vary between strains [24]. Because of their immunostimulatory properties, lactobacilli themselves are considered as potential vaccine adjuvants. For example, it has been shown that heat-killed *L. casei* functioned as an efficient adjuvant in combination with a nasal vaccine against *Steptococcus pneumoniae* [25]. In recent years, significant progress has been made in developing LAB as delivery vehicles for mucosal vaccines and therapeutic biomolecules [21, 26–29].

Despite three decades of massive research there is still no vaccine for human immunodeficiency virus type 1 (HIV-1) and the vaccine development remains a global priority. Protection against HIV-1 will likely depend on virus-specific antibodies, but a CD8^+^ T cells response, leading to elimination of infected cells, is also considered highly important [30]. The use of LAB as delivery host for a mucosal vaccine against HIV-1 has previously been shown to induce HIV-1 specific immune responses. Animal studies with an orally administrated *L. lactis* strain producing surface-anchored HIV-1 envelope protein induced effective and specific immunity in mice [31]. Likewise, *L. acidophilus* displaying the Gag antigen of HIV-1 elicited specific immune responses in vitro and in vivo [32].

In the present study, we have explored the possibility to express CCL3 together with a truncated HIV-1 Gag antigen in *L. plantarum* with the aim of increasing the recruitment of immune cells. We selected the Gag antigen since it is one of the most common and most immunogenic HIV-1 antigens [33–35] and because it was known that expression in *Lactobacillus* cells is possible and can lead to a specific immune response [32]. We fused CCL3 to Gag and exploited various *Lactobacillus plantarum*-derived surface anchoring domains in order to display the CCL3Gag fusion protein on the bacterial surface. Then we analyzed whether the fusion protein was correctly displayed at the exterior of the cell after which the chemotactic properties of bacteria producing CCL3Gag were investigated. We show that most of the recombinant strains indeed are able to recruit T cells in vitro and that biological activity of the chemotactic protein depends on the type of anchor used. To our knowledge, this is the first study showing the ability of an engineered *Lactobacillus* to produce a functional chemotactic protein immobilized on its surface.

## Results

### Construction of *L. plantarum* for display of the CCL3Gag fusion protein

**Fig. 1:**
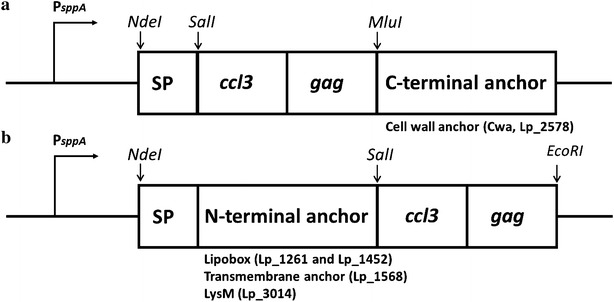
The expression cassette for C-terminal (**a**) and N-terminal (**b**) anchoring of CCL3Gag in *L. plantarum.* All parts of the cassette are easily exchangeable using restriction sites: *SalI* between the signal peptide or the N-terminal anchor and CCL3Gag, *EcoRI*, at the end of the insert, and *MluI* between CCL3Gag and the C-terminal anchor. **a** C-terminal anchoring was accomplished by fusing the N-terminus of the CCL3Gag fragment to a signal peptide (SP; from Lp_0373) and the C-terminus to a cell wall anchor (Cwa) from Lp_2578 (Cwa comprises 194 residues of Lp_2578) [42]. **b** Four N-terminal anchoring motifs were used, all containing an SP. Two lipoanchors were generated using lipobox fragments from Lp_1261 (residues 1–75) and Lp_1452 (residues 1–142), one transmembrane anchor was generated by fusing CCL3Gag to C-terminally truncated Lp_1568 (complete protein with 7-residues truncation), which contains an SP without a predicted signal peptide cleavage site, and one LysM anchor was generated by fusing CCL3Gag to full length Lp_3014 (a 204 residue putative transglycosylase with an N-terminal LysM domain)

Five different expression vectors were generated as described in the Materials and Methods section, with architectures outlined in Fig. 1. In all constructs, the Gag antigen was fused to the C-terminal end of CCL3, resulting in fusion protein CCL3Gag. CCL3Gag was linked to the bacteria via a C-terminal anchor (Fig. 1a) and four different N-terminal anchors (Fig. 1b). The C-terminal anchor was fused to the C-terminal end of CCL3Gag, consequently, CCL3 is expected to protrude from the bacteria after surface localization. For the constructs with N-terminal anchors, the Gag sequence forms the C-terminal part and is expected to protrude from the bacteria. The C-terminal anchor is a covalent cell wall anchor (Cwa) derived from Lp_2578 containing an LPxTG domain that ensures sortase-catalyzed covalent binding to peptidoglycan. We used four N-terminal anchoring sequences of different nature: (1) a non-covalent anchor derived from Lp_3014 encoding a LysM domain with affinity for peptidoglycan, (2) two lipoprotein anchors derived from Lp_1261 and Lp_1452 which attach the target protein covalently to the cell membrane, and (3) a transmembrane anchor (a non-cleaved signal peptide) derived from Lp_1568. *L. plantarum* transformed with pEV, an empty plasmid lacking the sequence encoding CCL3Gag [36], was used as negative control (referred to as *Lp*_Ev).

### Production of anchor-fused CCL3Gag protein

**Fig. 2:**
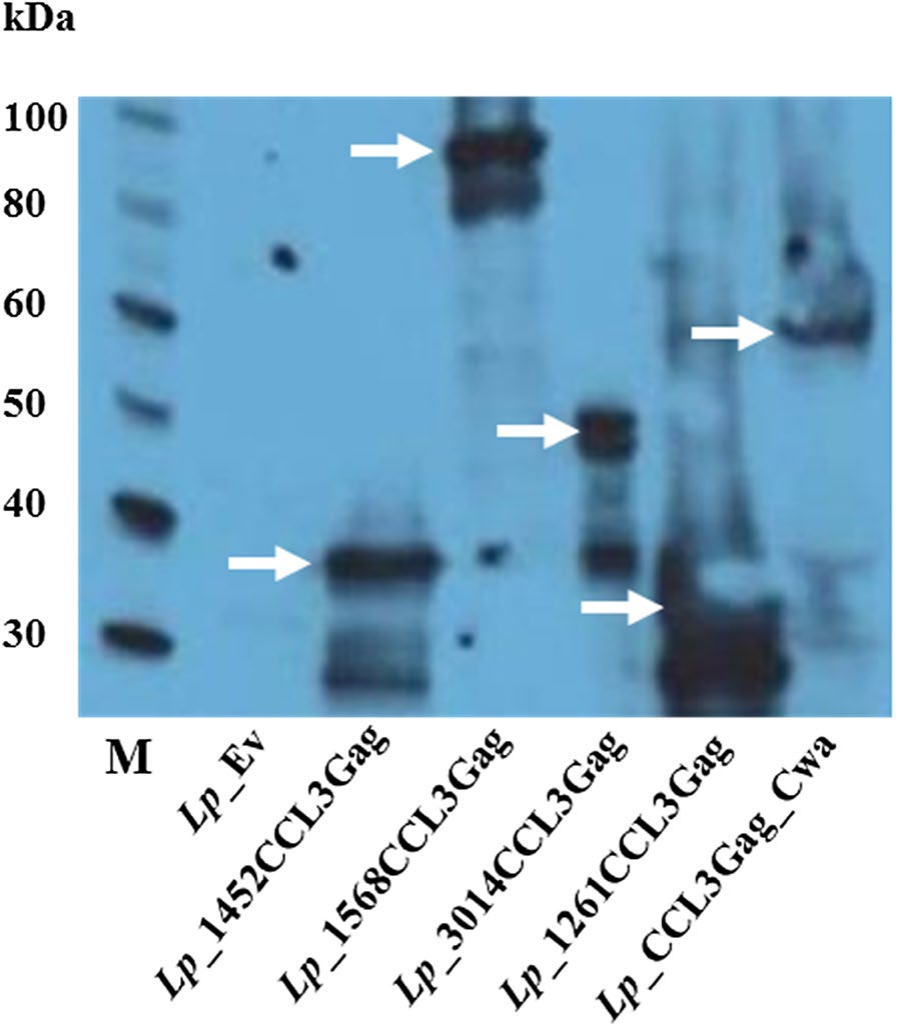
Detection of CCL3Gag fusion protein produced in *L. plantarum* strains harboring various plasmids. Bacterial cell-free protein extracts were prepared 3 h after induction by use of peptide pheromone, and analyzed by Western blotting, using polyclonal goat anti-CCL3 antibody and polyclonal rabbit anti-goat HRP-conjugated IgG. A strain harboring the pEV plasmid [36], not containing the *ccl3gag* fragment, was used as a negative control. The following CCL3Gag producing strains were analyzed (expected mass of the CCL3Gag protein between parenthesis): *Lp*_1452CCL3Gag (39.9 kDa), *Lp*_1568CCL3Gag (95.3 kDa), *Lp*_3014CCL3Gag (45.0 kDa), *Lp*_1261CCL3Gag (31.9 kDa), *Lp*_CCL3Gag_Cwa (50.5 kDa). The arrows indicate the location of the fusion proteins. Lane M shows a molecular mass standard

Expression of the gene encoding the hybrid proteins was induced by adding a peptide pheromone to the growing cultures of the recombinant *L. plantarum* strains. In order to examine whether CCL3Gag was produced, crude protein extracts from induced strains were subjected to Western blotting using an anti-CCL3 antibody. The fusion of CCL3 and Gag has a molecular weight of 22 kDa, whereas the sizes of the anchors vary. Figure 2 shows bands with the expected masses for all CCL3Gag producing strains. In some of the extracts, putative breakdown products are also visible. As expected, no bands were observed in the protein extract of the negative control. Thus, the hybrid proteins were successfully produced by *L. plantarum*.

### Growth characteristics of CCL3Gag-expressing *L. plantarum* strains

**Fig. 3:**
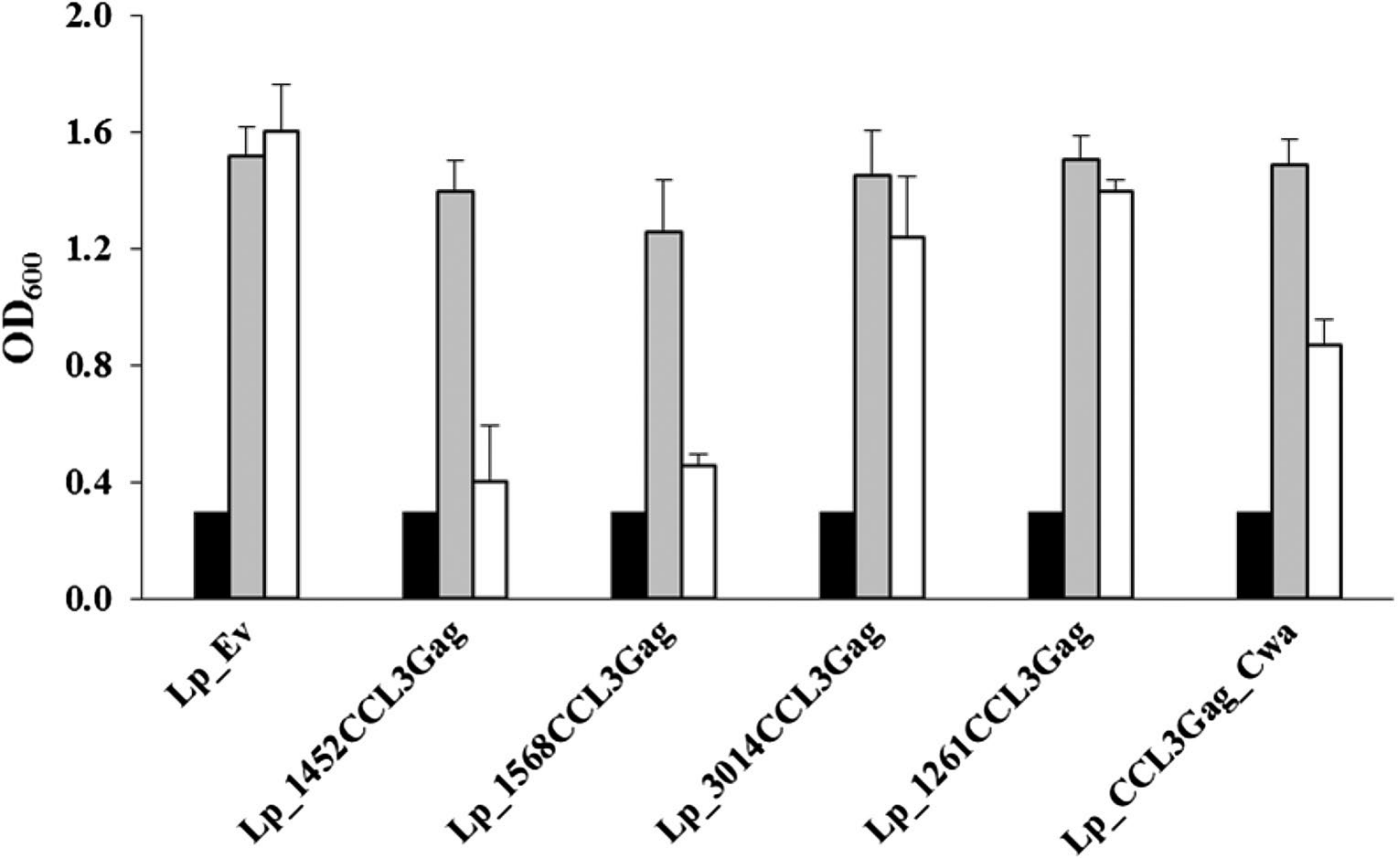
Growth of CCL3Gag-expressing *L. plantarum* cells. OD_600_ values were measured at the induction point (*black bars*). Next, each culture was divided into a pheromone induced culture (*white bars*) and a non-induced culture (*gray bars*). OD_600_ values were measured 3 h after the point of induction. The data are presented as the means from triplicates +SD

We investigated whether expression and secretion of the heterologous proteins may be stressful and reduce the growth of the recombinant *L. plantarum*. Figure 3 shows that the non-induced recombinant strains engineered for CCL3Gag production had similar growth rates as the strain containing the empty vector. Upon induction, clear growth retardation was observed for strains harboring pLp_1452CCL3Gag, pLp_1568CCL3Gag, and, to a lesser extent, pLp_CCL3Gag_Cwa. The growth of induced strains harboring pLp_3014CCL3Gag or pLp_1261CCL3Gag differed only slightly from the growth of the non-induced and control cultures (Fig. 3). Interestingly, the effect of induction on growth was dependent on the anchor used and the growth effects observed here show similar tendencies as in a previous study using the same anchoring sequences [36]. Notably, all strains did grow upon induction and Fig. 2 shows that the CCL3Gag was produced.

### Surface display of CCL3 in *L. plantarum*

**Fig. 4:**
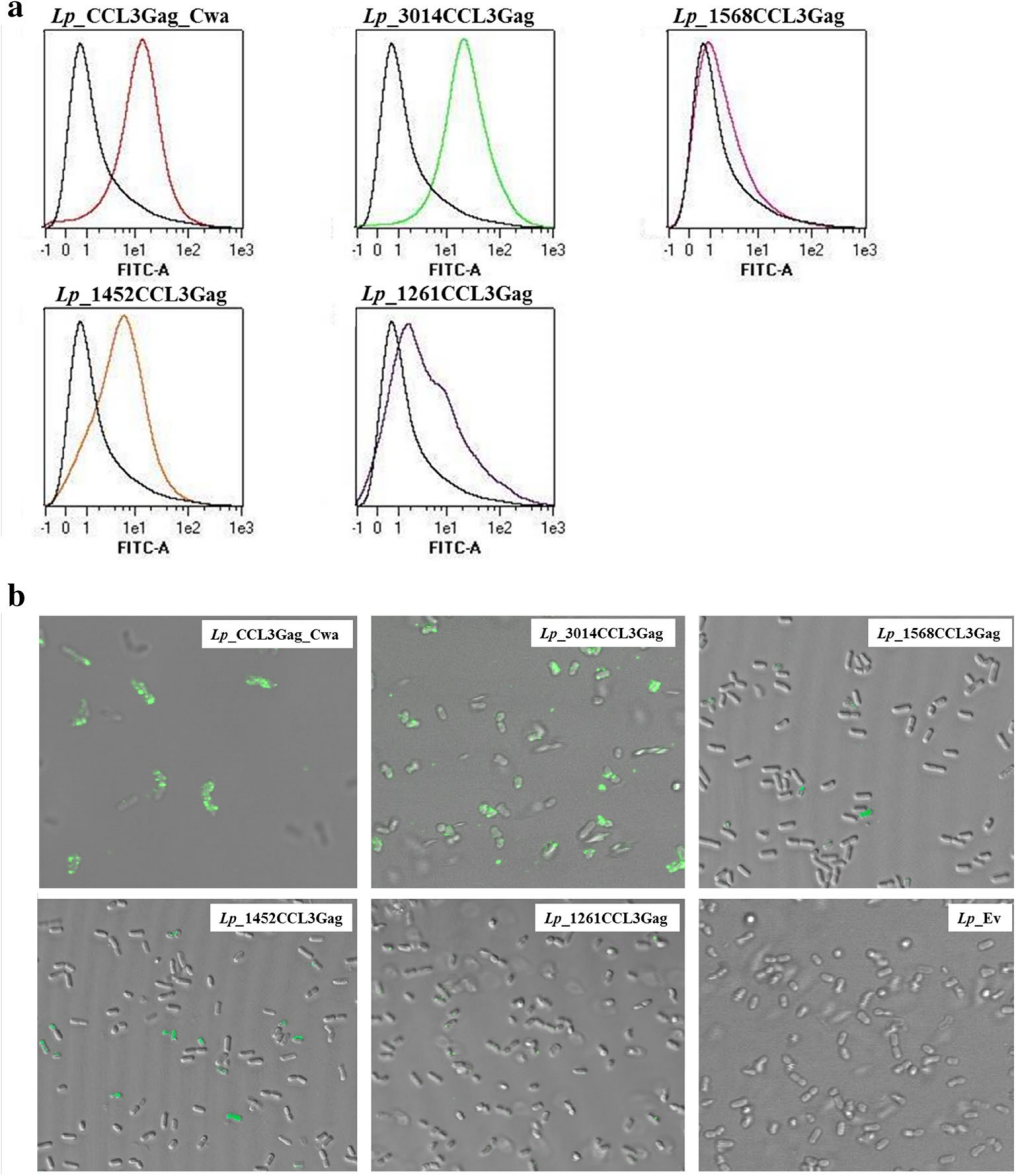
Flow cytometry (**a**) and microscopy (**b**) analysis of surface localization of CCL3Gag*. L. plantarum* cells harboring plasmids designed for N- or C-terminal anchoring of CCL3Gag were probed with goat anti-CCL3 polyclonal antibody and, subsequently, Alexa Fluor^®^488-conjugated rabbit anti-goat IgG antibodies. *L. plantarum* harboring pEV without the *ccl3gag* gene fragment was used as a negative control and is shown in all five histograms in panel **a** (*black line*). The data are presented as one representative experiment. Each experiment was performed at least three independent times and gave the similar results

In order to confirm the surface localization of the CCL3Gag protein, we carried out flow cytometry analysis and fluorescence microscopy, by labeling of live bacteria with an anti-CCL3 antibody (Fig. 4). The flow cytometry analysis showed a clear increase of fluorescence intensity for *Lp*_CCL3Gag_Cwa, *Lp*_3014CCL3Gag, *Lp*_1452CCL3Gag and *Lp*_1261CCL3Gag, compared to the negative control, suggesting surface localization of the CCL3Gag protein (Fig. 4a). Notably, the results show substantial differences between the individual strains. For the strain producing the transmembrane-anchored version of CCL3Gag (*Lp*_1568CCL3Gag), the fluorescence intensity was not substantially different from the negative control (Fig. 4a). Fluorescence microscopy (Fig. 4b) showed the strongest signals for strains in which CCL3Gag is attached to the cell wall (*Lp*_CCL3Gag_Cwa and *Lp*_3014CCL3Gag). These strains also gave the highest fluorescence intensity in flow cytometry (Fig. 4a). Interestingly, we observed differences in fluorescence intensity for the two strains producing CCL3Gag with lipoprotein anchors. Both in flow cytometry and in fluorescence microscopy *Lp*_1452CCL3Gag showed higher fluorescence intensity compared to *Lp*_1261CCL3Gag.

### Effect of surface displayed CCL3 on chemotaxis

To determine whether CCL3 showed biological activity when fused to Gag and surface-attached by *L*. *plantarum*, we tested the bacteria in a chemotaxis assay with a murine T cell line. The cell line is denoted Esb-MP and expresses the CCL3 receptors CCR1 and CCR5 [37, 38]. As positive and negative controls, we used free recombinant murine CCL3 and *Lp*_Ev, respectively.

**Fig. 5:**
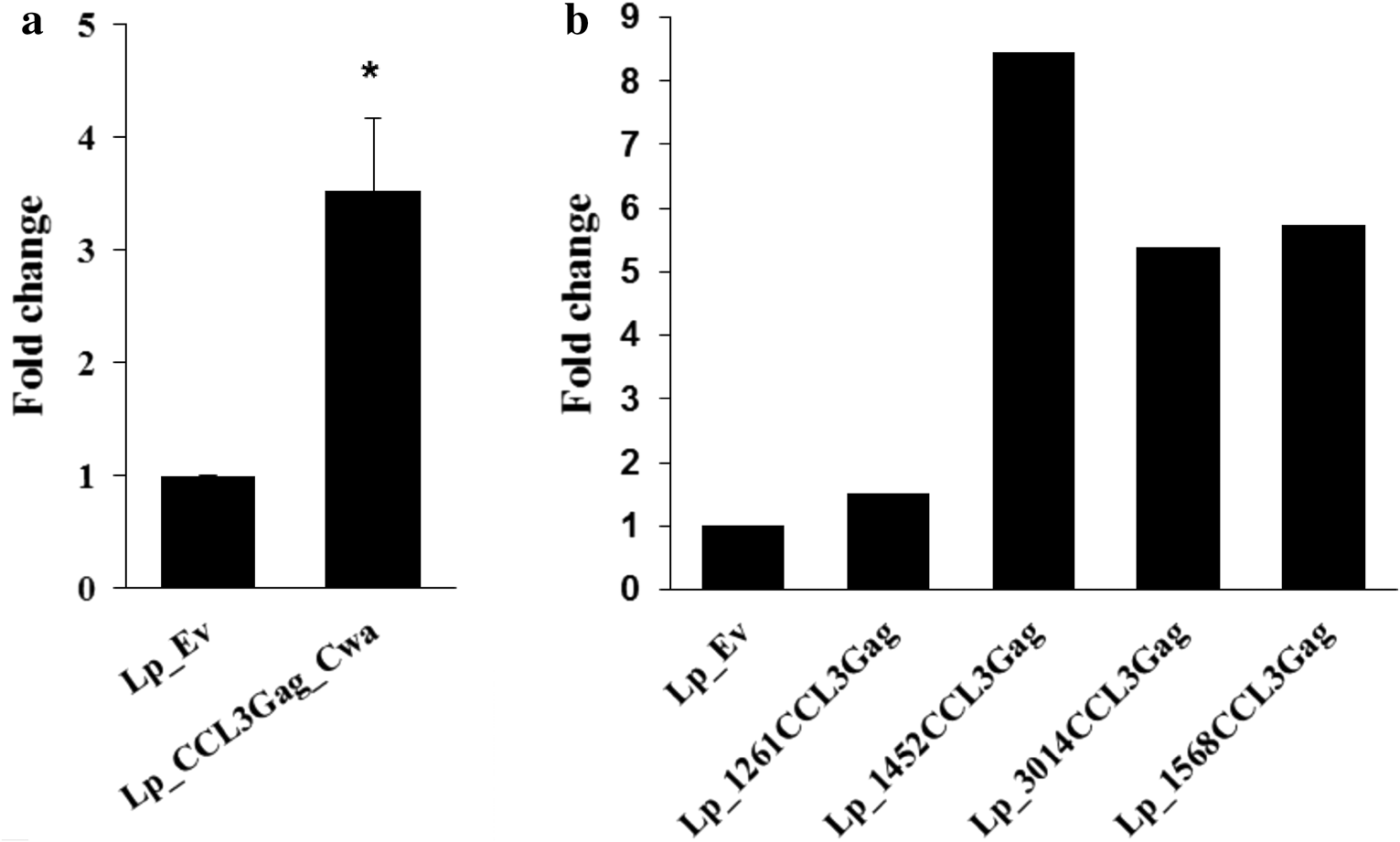
Chemotactic properties of *L. plantarum* harbouring various constructs. The *graphs* show migration of Esb-MP cells towards CCL3Gag-expressing strains compared to migration towards the negative control (*Lp*_Ev). The number of migrated cells was counted using flow cytometry and relative chemotaxis is shown as the average fold change relative to the negative control. **a** *L. plantarum* strain displaying CCL3Gag anchored C-terminally to the surface. The data presented are the means from 3 replicates +SEM. Statistically significant differences compared to the negative control (*p* < 0.01) are indicated by an *asterisk* (*). **b** *L. plantarum* strains displaying CCL3Gag anchored N-terminally to the surface. The data presented are derived from one representative experiment. The experiments were performed at least three times and these independent experiments showed the similar trends

In an initial experiment, we examined the chemotactic activity of *Lp*_CCL3Gag_Cwa strain, in which CCL3Gag is covalently attached to the cell wall, i.e. the most outer part of the bacteria, and in which the N-terminal domain of CCL3, essential for chemotaxis activation, is likely to be the most external part of the surface-coupled protein. Figure 5a shows that this strain indeed was able to attract T cells. The number of migrated cells towards bacteria displaying CCL3 was nearly fourfold higher compared to the negative control strain. This result shows that CCL3 localized on the surface of *L. plantarum* is biologically active.

In the next step, we tested chemotactic functionality of the four strains producing CCL3Gag protein fused to N-terminal anchors. These experiments suffered from rather large variations in the counts of migrated cells, but did consistently indicate that all strains, except *Lp*_1261CCL3Gag, promote increased recruitment of Esb-MP cells relative to the negative control (Fig. 5b). The variations between independent experiments were too high to determine statistical significance. The fact that *Lp*_1261CCL3Gag showed the lowest levels of surface accessible protein (Fig. 4) and caused no chemotaxis (Fig. 5b) may be taken to add confidence to the data. On the other hand, however, *Lp*_1568CCL3Gag did consistently cause chemotaxis, whereas no signals were obtained in flow cytometry and fluorescence microscopy.

**Fig. 6:**
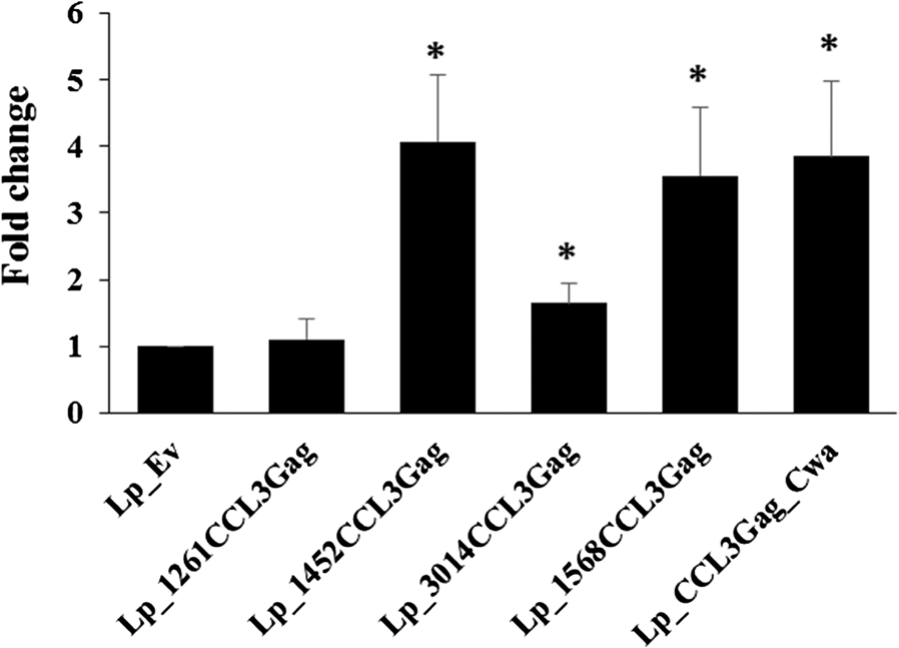
Chemotactic properties of *L. plantarum* strains surface displaying CCL3Gag, in presence of soluble CCL3 protein. Migration of Esb-MP cells towards CCL3Gag expressing strains in the presence of 3 ng/ml free CCL3 was compared to migration towards the negative control (*Lp*_Ev). The number of migrated cells was counted using flow cytometry and relative chemotaxis is shown as average fold change relative to the negative control. The data are presented as the means from at least 5 replicates +SEM. Statistically significant differences (*p* < 0.01) are indicated by an *asterisk* (*)

The chemotactic activity of surface-displayed CCL3, particularly when N-terminally anchored, may be hampered by steric constraints caused by the fusion with an anchor and Gag, and/or by a cell wall embedded localization masking interacting regions of the protein. Likewise, the oligomerization of CCL3, which contributes to activity [7], is likely to be restricted by the surface immobilization. We hypothesized that addition of soluble CCL3 in amounts that are too low to cause full chemotaxis could enhance chemotaxis caused by the CCL3Gag-displaying bacteria. Therefore, we carried out chemotaxis experiments where we added low concentrations (3 ng/ml) of CCL3 to bacteria expressing CCL3Gag and to the negative control strain. As expected, the presence of free CCL3 in the bacterial suspension led to cell migration for all strains (including the negative control; data not shown). There were, however, significant differences in degree of chemotaxis between the negative control and all CCL3Gag-producing strains, except *Lp*_1261CCL3Gag (Fig. 6). For three strains, *Lp*_1452CCL3Gag, *Lp*_1568CCL3Gag and *Lp*_CCL3Gag_Cwa, the Esb-MP migration was approximately fourfold higher (compared to *Lp*_Ev), whereas the fold change for *Lp*_3014CCL3Gag was below two.

To exclude positive or negative effects of *L. plantarum* itself on chemotaxis, we performed the chemotaxis assay with control strain *Lp*_Ev in the absence and presence of the low concentration (3 ng/ml) of CCL3 and compared with a set-up without added bacteria at all (only T cell medium). The results showed that *L. plantarum* did not exhibit chemotactic properties in the absence of added chemokine (Additional file 1a). Moreover, the bacteria did not increase chemotaxis induced by 3 ng/ml CCL3 (Additional file 1b), confirming a lack of natural chemotactic properties.

## Discussion

The aim of this study was to analyze the functionality of a chemotactic protein displayed on the surface of *L. plantarum* cells. Surface-display of functional chemokines may eventually lead to development of more effective LAB-based mucosal vaccines. The results show that CCL3Gag was successfully expressed in *L. plantarum* (Fig. 2), that the protein can be detected at the bacterial surface in most recombinant strains (Fig. 4), and that expression of CCL3Gag indeed promotes chemotaxis (Figs. 5, 6). Production of secreted functional chemokines in LAB has been demonstrated before [39–41]. What is new here is that we used *Lactobacillus*, which has a considerably stronger potential as vaccine delivery vehicle compared to *L. lactis*, the bacterium most often used in the studies published so far [22]. Furthermore, we used surface-display rather than secretion, because proteins immobilized on the cell surface are potentially more stable and more efficiently protected from degradation than the secreted protein.

In order to direct the CCL3Gag fusion protein to the surface of *L. plantarum*, we used four different types of anchors, previously used for surface display of Invasin from *Yersinia pseudotuberculosis* [36] and an oncofetal antigen [42] in *Lactobacillus*. Some prior studies showed that the functionality of heterologous proteins anchored to the surface of *Lactobacillus* strongly depends on the type of anchor used. For example, Invasin-expressing *L. plantarum* strains activated NF-κB expression in monocytes to varying levels depending on the anchoring methods. In this case the two lipo-anchors (from Lp_1261 and Lp_1452) worked best whereas the LysM-based anchor (from Lp_3014) did not work [36]. Likewise, two *L. acidophilus* strains displaying the *Salmonella* FliC protein with two different cell wall anchors showed clear differences in terms of their maturating effect on DCs and stimulation of cytokine production [43].

It is difficult to answer the question why different anchors give different results and why the functionality of the to-be-displayed functional protein depends on anchor types (note that conclusions on anchor functionality from the previous Invasin study differ from the conclusions of this study). For example, weak (*Lp*_1261CCL3Gag; lipoprotein anchor) or minimal (*Lp*_1568CCL3Gag; transmembrane anchor) signals in flow cytometry and microscopy may be due to lower protein production, a more embedded localization in the cell wall, or a sub-optimal orientation of the reactive part of the displayed protein. In functional studies, in particular in vivo studies, proteolytic susceptibility, and stability in general, are additional variables. In the two strains giving the lowest signal in flow cytometry, the CCL3 is located N-terminally, directly following the anchor (Fig. 1b). This location may prevent efficient interaction of the anti-CCL3 antibody with CCL3 epitopes. Interestingly, *Lp*_1452CCL3Gag gave much stronger signals than the other lipoanchor strain, *Lp*_1261CCL3Gag; in the former strain, the anchor sequence is 67 residues longer, which could make the CCL3 domain more exposed and accessible to the antibody.

The results of the chemotaxis assays were largely consistent with the results from the flow cytometry and microscopy analyses. Convincing data were obtained for *Lp*_CCL3Gag_Cwa, which gave the strongest signal in immunofluorescence detection and was clearly able to recruit murine T cells in vitro (Fig. 5a). Interestingly, in this strain the N-terminal domain of CCL3, which is essential for interaction with the CCL3 receptor [6, 44, 45], is likely to be highly exposed (Fig. 1a). In the four remaining strains, the N-terminal chemokine domain is likely to be less exposed (Fig. 1b). The chemotaxis data for these strains varied between each experiment, but showed consistent trends, suggesting that all strains except *Lp*_1261CCL3Gag and the negative control induce chemotaxis.

Migration of chemoattracted cells is always preceded by receptor activation through direct interaction with the chemokine [6, 44]. Such receptor binding is often stimulated by formation of higher protein structures, such as dimers or oligomers [7, 8]. This process is likely hampered for surface anchored and possibly embedded CCL3Gag. We showed that free soluble CCL3 protein, added at a concentration that is clearly suboptimal for full activity (according to the supplier), promoted increased chemotaxis for *Lp*_CCL3Gag_Cwa, *Lp*_1452CCL3Gag, *Lp*_3014CCL3Gag, *Lp*_1568CCL3Gag relative to the negative control strain (Fig. 6). For the Lp_1261 lipoprotein anchor the fluorescence signal was weak and the strain did not induce chemotactic activity, which suggests either not sufficient surface display of CCL3 or that the displayed CCL3 lacks biological activity. Generally, in all experiments, we used similar amounts of bacterial cells, but it should be noted that the number of proteins at the surface might differ between the recombinant strains, which of course will influence strain functionality.

Importantly, the current results indicate that the translational fusion between CCL3 and the Gag antigen did not abolish the functionality of the chemokine. The biological activity of CCL3 fused to other proteins has been determined for fusions with a tumor antigen [46], HIV-1 gp-120 [17], mCherry fluorescent protein [47], and hemagglutinin from influenza virus [48]. In these prior studies, the fusion protein was tested in free soluble form, and not in an immobilized form as was done here. In this regard, it was important to check whether the observed chemotaxis really was an effect of protein immobilized on the bacterial cell, and not a result of free protein released as an effect of bacterial lysis and protein degradation, or by shedding from the surface. In a control experiment, we collected the cell-free supernatants from a chemotaxis experiment and analyzed these by Western blotting. Very weak bands were detected only for *Lp*_1261CCL3Gag and *Lp*_3014CCL3Gag, which are among the variants causing no or little chemotaxis (data not shown). Importantly, no soluble protein was detected in experiments with the best performing strain, *Lp*_CCL3Gag_Cwa. So, it seems that the observed chemotaxis is not due to released soluble protein, although this cannot be excluded for *Lp*_3014CCL3Gag.

## Conclusion

The present study shows that *L. plantarum* NC8 was able to produce a fusion protein consisting of a murine chemokine, CCL3, and a truncated HIV-1 Gag antigen, and to display the protein on the bacterial surface. Chemotaxis assays demonstrated that the CCL3 domain was functional, albeit to varying extents that depended on the type of anchor used. The fusion of the Gag antigen with CCL3 may constitute a valuable approach in development of a HIV-1 vaccine. Further work to verify the validity of this approach is currently considered.

## Methods

### Bacterial strains, plasmids and growth conditions

The bacterial strains and plasmids used in this study are listed in Table 1. *Lactobacillus plantarum* strains were cultured in MRS broth (Oxoid Ltd., Basingstoke, UK) at 37 °C without shaking. *Escherichia coli* TOP10 cells (Invitrogen, Carlsbad, CA, USA) were grown in Brain Heart Infusion (BHI, Oxoid) broth and incubated at 37 °C with shaking. Erythromycin was added to final concentrations of 10 µg/ml for *L.plantarum* and 200 µg/ml for *E. coli*. Liquid medium was solidified by adding 1.5 % (w/v) agar.

**Table 1:** Strains and plasmids used in this study

Strain or plasmid	Description	References
Plasmids		
pUC-CCL3Gag	Amp^r^; pUC57 vector with synthetic *ccl3gag* gene	Genescript
pEV	Ery^r^; control plasmid (“empty vector”)	[36]
pLp_1261Inv	Ery^r^; pLp_2588sAmyA [51] derivative, encoding a lipoanchor sequence from lp_1261 fused to a fragment of an invasin encoding gene; for anchoring through an N-terminal lipobox.	[36]
pLp_1452Inv	Ery^r^; as pLp_1261Inv, but with the lipoanchor from Lp_1452.	[36]
pLp_3014Inv	Ery^r^; pLp_2588sAmyA [51] derivative, encoding Lp_3014 fused to the a fragment of an invasin encoding gene; for anchoring through an N-terminal LysM domain.	[36]
pLp_1568InvS	Ery^r^; pLp_2588sAmyA [51] derivative, encoding Lp_1568 fused to a fragment of an invasion encoding gene; for N-terminal anchoring through a signal peptide-like transmembrane helix.	[36]
pLp_0373sOFAcwa2	Ery^r^; pLp_0373sNuc [51] derivative, encoding signal peptide sequence from Lp_0373 fused to the *ofa* gene and a subsequent anchor encoding sequence (*cwa2*); for C-terminal covalent anchoring to the cell wall.	[42]
pLp_1261_CCL3Gag	Ery^r^, pLp_1261Inv derivative, where *inv* gene fragment has been replaced by the *ccl3gag* fusion gene.	This study
pLp_1452_CCL3Gag	Ery^r^, pLp_1452Inv derivative, where *inv* gene fragment has been replaced by the *ccl3gag* fusion gene.	This study
pLp_3014_CCL3Gag	Ery^r^, pLp_3014Inv derivative, where *inv* gene fragment has been replaced by the *ccl3gag* fusion gene.	This study
pLp_1586_CCL3Gag	Ery^r^, pLp_1568InvS derivative, where *inv* gene fragment has been replaced by the *ccl3gag* fusion gene	This study
pLp_CCL3Gag_Cwa	Ery^r^, pLp_0373sOFAcwa2 derivative, where the *ofa* gene has been replaced by the *ccl3gag* fusion gene	This study
Strains		
*L. plantarum* NC8	Host strain	[55]
*E. coli* TOP10	Host strain	Invitrogen
*Lp*_1261CCL3Gag	*L. plantarum* NC8 harboring pLp_1261CCL3Gag; for surface display of the CCL3Gag protein using an N-terminal lipo-anchor	This study
*Lp*_1452CCL3Gag	*L. plantarum* NC8 carrying pLp_1452CCL3Gag; for surface display of the CCL3Gag protein using an N-termina lipo-anchor	This study
*Lp*_3014CCL3Gag	*L. plantarum* NC8 carrying pLp_3014CCL3Gag; for surface display of the CCL3Gag protein using an N-terminal LysM domain	This study
*Lp*_1568CCL3Gag	*L. plantarum* NC8 carrying pLp_1568CCL3Gag; for surface display of the CCL3Gag protein using an N-terminal transmembrane anchor	This study
*Lp*_CCL3Gag_Cwa	*L. plantarum* NC8 carrying pLp_CCL3Gag_Cwa; for surface display of the CCL3Gag protein using a C-terminal covalent cell wall anchor (Cwa)	This study
*Lp*_Ev	*L. plantarum* NC8 carrying pEV (emtpy vector); used as a negative control strain	[36]

### DNA manipulations and plasmid construction

The primers used in this study are listed in Table 2. All expression plasmids used in this study are derivatives of the pSIP400 vector, constructed and developed for inducible gene expression, secretion and surface anchoring of proteins in *Lactobacillus* spp [42, 49–51]. For practical reasons, pSIP400 derivatives encoding a bacterial invasin [36] or oncofecal antigen [42] fused to the diverse anchors were used as starting point for cloning. The gene construct for the fusion protein was such that the C-terminal end of CCL3 [GenBank: NP_035467] was fused to an antigen derived from group specific antigen (Gag) of HIV-1 isolate HXB2 [52] without a linker. The antigen part in this study is denoted Gag and covered amino acids 192–315 of Gag [GenBank: AAB50258.1], which comprises major determinants of immunogenicity including the MHC class I (H-2 Kd)-restricted peptide AMQMLKETI. The *ccl3gag* gene fragment was codon optimized for expression in *L. plantarum,* synthesized at Genscript (Piscataway, NJ) and cloned into a pUC57 plasmid, yielding pUC-CCL3Gag. The pUC-CCL3Gag plasmid was digested with *SalI* and *EcoRI* and the *ccl3gag* fragment was ligated into *SalI/EcoRI* digested pLp_1452Inv, pLp_1261Inv and pLp_3014Inv plasmids, yielding the plasmids pLp_1452CCL3Gag, pLp_1261CCL3Gag and pLp_3014CCL3Gag, respectively. A sequence encoding the N-terminal part of Lp_1568 was amplified from pLp_1568Inv, using primers 1568F and 1568R, and inserted to pLp_1261CCL3Gag, digested with *NdeI* and *SalI*, using In-Fusion^®^HD Cloning Kit (Clontech Laboratories, Mountain View, CA) yielding the plasmid pLp_1568CCL3Gag. Plasmid pLp_0373sOFAcwa2 was digested with *SalI* and *MluI* and ligated by In-Fusion cloning to a *ccl3gag* fragment amplified from pUC–CCL3Gag using primers cclF and cclR, yielding the plasmid pLp_CCL3Gag_Cwa.

**Table 2:** Primers used in this study

Primer	Sequence
1568F	GGAGTATGATTCATATGAAATTGTTTAAGAAAATTACGAT^a^
1568R	CGTATGGGGCGTCGACCGCTGCATAAATTTGCTTAGCAAC^a^
cclF	TGCTTCATCAGTCGACGCCCCATACGGTGCT^b^
cclR	GTTCAGTGACACGCGTGTTCTTGACTTCTTGACTCGCTTGT^b^

^a^Underlining indicates 15-bp extensions that are complementary to the ends of the *NdeI/SalI*-digested pLp_1261CCL3Gag vector. Overlapping sequences are necessary when using In-fusion cloning

^b^Underlining indicates 15-bp extensions that are complementary to the ends of the *SalI/MluI*-digested pLp_0373sOFAcwa2 vector

All plasmids were first transformed into *E. coli* TOP10. Positive clones were screened by PCR, restriction enzyme digestion and sequenced. Purified plasmids were electroporated into *L. plantarum* cells according to Aukrust et al. [53].

### Harvesting of strains

Overnight cultures of *L. plantarum* strains harboring plasmids carrying the *ccl3gag* fusion gene were diluted in fresh MRS medium to OD_600_ ~0.1 and incubated at 37 °C until the OD_600_ reached ~0.3. Recombinant CCL3Gag expression was then induced by adding the peptide pheromone to a final concentration of 25 ng/ml [54]. Bacterial cells were harvested 3 h after induction, unless otherwise indicated, by centrifugation at 5000×*g*, 4 °C, for 5 min. Pellets were washed twice with Phosphate Buffered Saline (PBS) before using in further experiments. In order to determinate the number of colony forming units (CFU), harvested bacterial cells were cultivated on solid MRS medium supplemented by antibiotic for 24 h and the colonies were counted.

### Western blotting

To analyze CCL3Gag expression, bacterial cells were harvested from 50 ml of cultures, resuspended in 1 ml PBS and added to FastPrep tubes containing glass beads (Sigma-Aldrich). Cell-free protein extracts were prepared by disruption in a FastPrep^®^ FP120 Cell Disrupter by shaking at a speed of 6.5 m/s for 45 s. Cell debris was removed by centrifugation at 12,000×*g*, 4 °C, for 2 min. Proteins were separated by SDS–polyacrylamide gel electrophoresis and transferred to a nitrocellulose membrane using the iBlot™ Dry Blotting System (Invitrogen). Proteins were detected using the SNAP i.d.^®^ 2.0 Protein Detection System (Merck kGaA Darmstadt, Germany) using a specific polyclonal goat anti-CCL3 antibody (R&D Systems, BAF450), 1:10,000 and, subsequently, a polyclonal rabbit anti-goat HRP-conjugated (Abcam) antibody, diluted 1:5000.

### Flow cytometry and indirect immunofluorescence microscopy of *L. plantarum* expressing CCL3Gag

Bacterial strains were harvested as described above. Approximately 1 × 10^7^ CFU were resuspended in 50 µl PBS containing 2 % (w/v) bovine serum albumin (BSA, Sigma-Aldrich) and 0.4 µl polyclonal goat anti-CCL3 antibody and incubated for 30 min at room temperature. After incubation, cells were centrifuged at 5000×*g* for 5 min and washed 4 times with PBS containing 2 % BSA. Subsequently, cells were resuspended in 50 µl PBS containing 2 % BSA and polyclonal donkey Alexa Fluor^®^488 conjugated anti–goat antibody (Molecular Probes, Life technologies, USA) followed by incubation in darkness and at room temperature for 30 min. Cells were collected by centrifugation, washed 4 times with PBS, and resuspended in 100 µl PBS without BSA. The bacterial suspensions were immediately analyzed by flow cytometry using a MACSQuant analyzer (Miltenyi Biotec GmbH, Bergisch Gladbach, Germany), following the manufacturer’s instructions. For indirect immunofluorescence microscopy, the bacteria were visualized under a Leica TCS SP5 Confocal laser scanning microscope (CSLM) using a 488-nm argon laser for the Alexa Fluor^®^488 photomultiplier tube (PMT) and bright field PMT for transmitted light.

### Cell lines

The T-lymphoma cell line Esb-MP was kindly provided by Prof. Schirrmacher, (Deutsches Krebsforschungszentrum, Heidelberg, Germany). The cells were cultured in DMEM medium (Gibco) supplemented with 10 % fetal bovine serum (FBS) and antibiotics (Penicillin–Streptomycin, Sigma-Aldrich). Cells were maintained in a humidified incubator at 37 °C and 5 % CO_2_.

### CFSE staining

5 mM EDTA in PBS or 0.25 % Trypsin–EDTA (Sigma-Aldrich) was used to detach the Esb-MP cells from the culture flasks (both detachment methods worked equally well). The cells were collected by centrifugation at 250 g for 8 min at room temperature and washed twice with PBS. Subsequently, cells were incubated in PBS with 5 µM carboxyfluorescein succinimidyl ester (CFSE; CellTrace™ CFSE Cell Proliferation Kit, Molecular Probes, Life Technologies) for 30 min at 37 °C, protected from light. Stained cells were washed and resuspended in RPMI 1640 medium (Gibco) containing 10 % (v/v) FCS without antibiotics.

### Chemotaxis assay

Approximately 1 × 10^8^ CFU where used when testing the chemotactic abilities of CCL3Gag-expressing *L. plantarum.* The bacterial pellets, harvested 3 h after induction as described above and were either tested in chemotaxis assay the same day or were stored at 4 °C overnight (no differences in chemotaxis were observed upon storage). The bacterial pellets were resuspended in 600 µl RPMI 1640 medium (Gibco) containing 10 % FCS and 20 mM HEPES buffer and placed in the lower wells of 24-well 6.5 mm Transwell plates with 0.5 μm pore size polycarbonate membrane filters (Transwell^®^ Permeable Supports; 5.0 µm polycarbonate membrane; 6.5 mm inserts, Corning Incorporated, Corning, NY, USA). In some experiments, we modified the chemotaxis assay by adding recombinant murine CCL3 (R&D Systems) to the bacterial suspensions to final concentrations of 3 ng/ml, that were lower than those needed to achieve full chemotactic activity, in order to initiate and enhance chemotaxis. CCL3 at a concentration of 25 ng/ml in RPMI 1640 medium with FBS was used as positive control. 2 × 10^6^ CFSE-labeled Esb-MP cells, resuspended in 100 µl RPMI 1640 medium with FCS were added to the inserts. The plates were incubated in a humidified incubator at 37 °C for 2 h. Cells that migrated through the membrane were harvested from the lower wells and counted for 5 min by fluorescence-activated cell sorting (FACS). Alternatively, the total volumes (600 µl) harvested from the lower wells and containing migrated cells were counted using MACSQuant analyzer, following the manufacturer’s instructions.

### Statistical analysis

Quantitative experimental data come from replicate (>3) experiments and are presented as the means + standard deviations (SD) or standard error (SEM). Where relevant, the statistical significance of differences (*P* < 0.05) was determined by using a paired *t* test.

## Additional file

10.1186/s12934-015-0360-z This file shows the influence of *L. plantarum* on increasing (a) and reduction (b) of the amount of migrated cells. (A) Migration of Esb-MP cells towards RPMI-1640 medium alone and RPMI-1640 medium containing *L. plantarum* harboring pEV (*Lp_*Ev). (B) Migration of Esb-MP cells towards RPMI-1640 medium supplemented with 3 ng/ml CCL3, and towards RPMI-1640 medium containing *L. plantarum* harboring pEV and supplemented with 3 ng/ml CCL3. The total number of migrated cells was counted by flow cytometry. The presented data are from one representative experiment. The experiment was performed at least three independent times, with similar results.
