# Supplementary figures and images for: Lactobacillus plantarum displaying CCL3 chemokine in fusion with HIV-1 Gag derived antigen causes increased recruitment of T cells
Source: Microb Cell Fact. 2015 Oct 22;14:169. doi: 10.1186/s12934-015-0360-z (PMC4618854; doi:10.1186/s12934-015-0360-z)

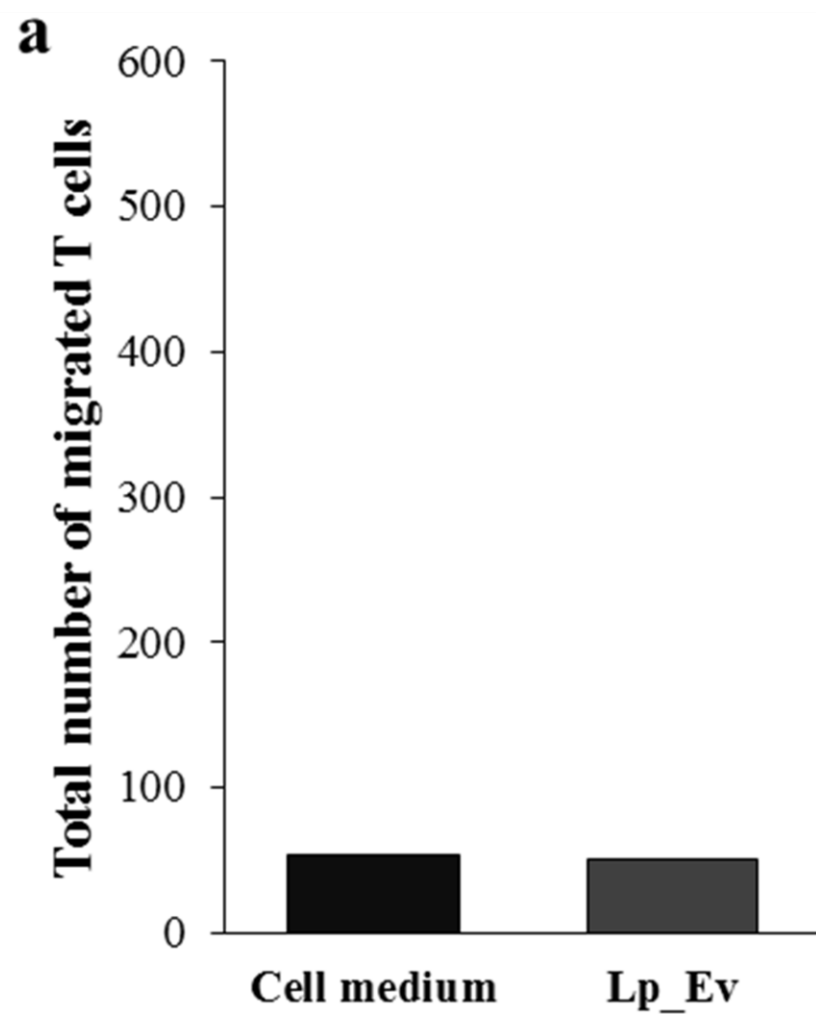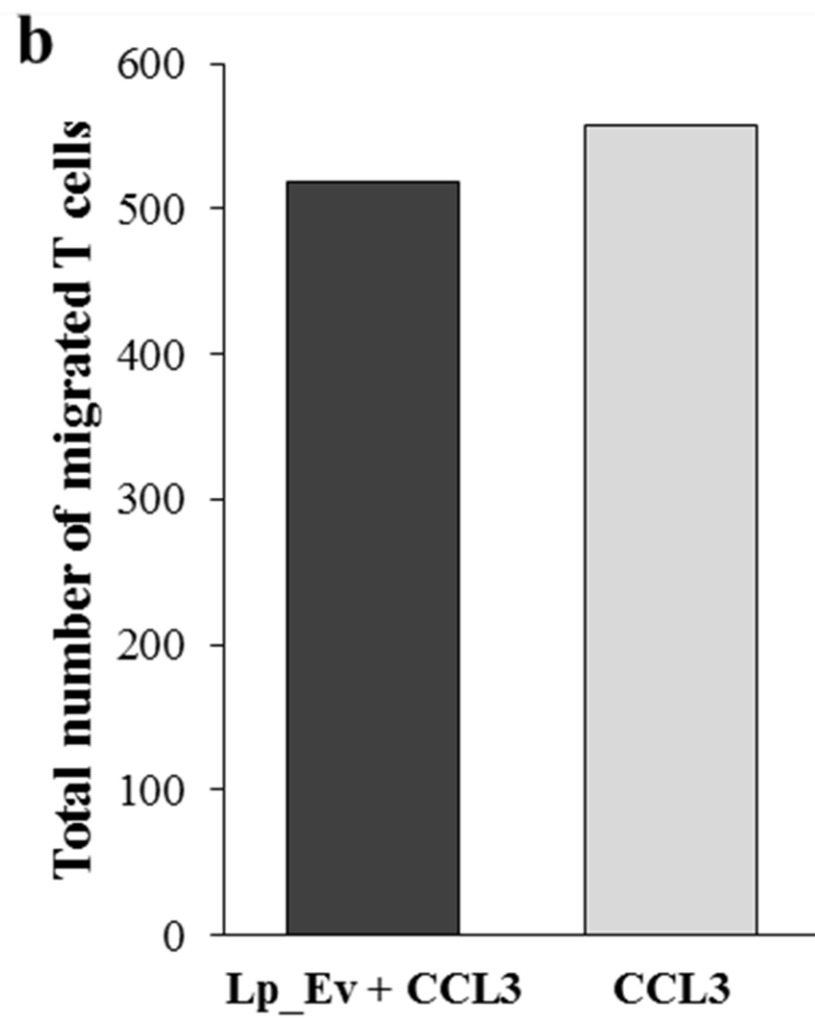

Supplement: Supplementary file 1 — 10.1186/s12934-015-0360-z This file shows the influence of L. plantarum on increasing (a) and reduction (b) of the amount of migrated cells. (A) Migration of Esb-MP cells towards RPMI-1640 medium alone and RPMI-1640 medium containing L. plantarum harboring pEV (Lp_Ev). (B) Migration of Esb-MP cells towards RPMI-1640 medium supplemented with 3 ng/ml CCL3, and towards RPMI-1640 medium containing L. plantarum harboring pEV and supplemented with 3 ng/ml CCL3. The total number of migrated cells was counted by flow cytometry. The presented data are from one representative experiment. The experiment was performed at least three independent times, with similar results. [file 12934_2015_360_MOESM1_ESM.pdf]
